# Metastatic Colon Cancer to the Thyroid Gland in the Setting of Pathologically Diagnosed Papillary Thyroid Cancer: A Review and Report of a Case

**DOI:** 10.4021/wjon276w

**Published:** 2011-02-26

**Authors:** Lee F. Starker, Flavio Paterno, Peyman Bjorklund, Dennis Wasson, Nabil Atweh

**Affiliations:** a Department of Surgery, Yale School of Medicine, New Haven CT, USA; b Department of Surgery, Bridgeport Hospital, Bridgeport CT, USA

**Keywords:** Thyroid cancer, Thyroid metastases, Colonic metastases, Papillary cancer

## Abstract

Colon carcinoma metastases to the thyroid are a rare phenomena. Here we report a case of multiple malignant neoplasms where an incidental diagnosis of colon cancer was made after pathologic evaluation of the thyroid specimen.

## Introduction

Thyroid cancer has previously been identified as a primary disease. Autopsy studies have demonstrated that metastatic neoplasms to the thyroid are common [1]. The incidence of metastatic disease in autopsy specimens has been reported to range from 1.25% up to a staggering 24% in a selected patient population who expired from widely metastatic disease [2-5]. Although metastatic disease can be present in various settings within the thyroid gland, these metastatic foci only account for 2% - 3% of all clinical cases of thyroid cancer [6]. Autopsy studies provide us with new evidence that a multitude of neoplasms metastasize to the thyroid gland including renal cell, lung and breast carcinomas. Colonic cancer metastases to the thyroid gland have been reported, however with a decreased frequency [5, 7, 8]. The incidence of such metastases does not seem to be common. We review a case of metastatic colon carcinoma to the thyroid within a cytologically and histopathologically diagnosed papillary thyroid cancer.

## Case Report

A 66-year-old Caucasian male presented with a palpable, painless slowly growing mass in the left side of the neck. He denied any local or systemic symptoms of thyroid dysfunction or neck compression. He did not report any history of radiation to the neck. His past medical history was remarkable for hypertension, hypercholesterolemia, and benign vocal cord polyps. He was taking verapamil, hydrochlorothiazide, atorvastatin and aspirin. He admitted to smoking 2 packs of cigarettes a day for 40 years and was a habitual alcohol user. Clinical examination revealed a firm, palpable, nontender nodule in the left lobe of the thyroid gland measuring 2.5 cm in diameter. The nodule was mobile upon swallowing. No palpable cervical nodes were appreciated. The rest of physical exam was unremarkable.

A thyroid ultrasound demonstrated multiple thyroid nodules within both thyroid lobes. The largest was in the left lobe, measuring 2.5 x 2.1 x 1.1 cm, and was hypoechoic and heterogeneous in nature. Fine-needle aspiration cytology was consistent with papillary thyroid carcinoma. Thyroid function tests at that time were normal.

**Figure 1:**
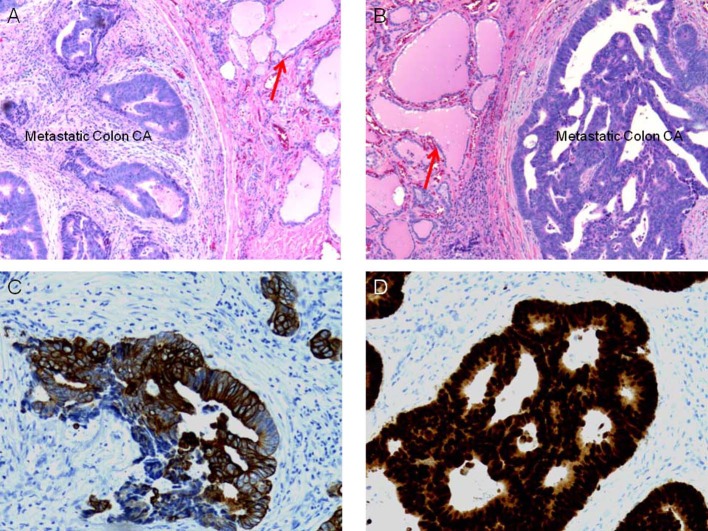
*Histological and immunophenotypical* examination of the resected specimen. (a) Hematoxilin and Eosin staining of the specimen, with clear delineation of native thyroid tissue and metastatic neoplastic disease, thyroid colloid depicted by red arrow. (b) Increased magnification of (a), again with red arrow depicting native thyroid colloid. (c) Immunohistological staining of specimen with anti-CK20 antibodies and (d) with anti-CDX-2 antibodies, suggesting the diagnosis of metastatic colon cancer to the thyroid.

The patient underwent an uncomplicated total thyroidectomy. The surgical and postoperative courses were unremarkable. Pathological examination confirmed a multifocal papillary thyroid carcinoma, involving both lobes and the isthmus. The surgical margins were negative. One anterior cervical (Delphian) lymph node was positive for metastatic disease (stage pT3N1a, stage III). A focus of metastatic adenocarcinoma was identified in the right lobe, characterized by glandular formation with central necrosis and hyperchromatic malignant cells. The histological and immunophenotypical features of this second tumor were consistent with adenocarcinoma of the colon (Fig. 1).

After this finding, the patient underwent a colonoscopy which revealed a mass in the descending colon consistent with adenocarcinoma. A Computed Tomography (CT) scan of the abdomen and pelvis revealed an 8 cm mass in the upper pole of the right kidney consistent with renal cell carcinoma. The patient underwent a left hemicolectomy, and a right nephrectomy. During this surgery a solitary 1.5 cm metastasis in the left lobe of the liver (segment III), was identified and resected. Pathologic examination confirmed the diagnosis of a colonic adenocarcinoma without nodal metastasis in the 31 nodes isolated (pT4N0M1), liver metastasis having a colonic origin with negative margins, and renal cell carcinoma (pT2N0).

After surgery the patient underwent radioactive iodine treatment and chemotherapy. He was disease-free for three years. After three years, the patients Carcinoembrionic Antigen (CEA) levels increased to 76.1 ng/ml (upper limit 5 ng/ml), with concomitant normal thyroglobulin levels. A CT scan of the abdomen and pelvis revealed a 3 cm mass in the left lobe of the liver. A Positron emission tomography (PET) scan confirmed a single area of abnormal tracer accumulation in the left lobe of the liver. He underwent an uneventful left hepatectomy. The lesion contained histopathologically proven metastatic colonic carcinoma with negative margins.

## Discussion

Colonic cancer is the third most common cancer in the United States today [9]. The usual route of metastatic disease is via the portal system and as such the liver is the most common organ for secondary disease. Rectal cancers however have a propensity to bypass the liver and directly seed the pulmonary parenchyma due to the systemic drainage of the rectal veins. Documented metastases from the colon and rectum to the thyroid are extremely rare.

Thyroid cancer is the seventh most common cancer in United States with the overwhelming majority of cases consisting of papillary thyroid cancer. This type of cancer has an exceptionally high survival rate, approaching 97% with surgical resection and careful follow-up for the presence of local recurrence within the thyroid bed [10]. Although gross metastatic disease to the thyroid remains uncommon, microscopic metastases are frequently detected in autopsy studies [11]. An overwhelming majority of metastasis to the thyroid arise from renal cell carcinomas, followed by lung and breast neoplasms [4, 8]. The majority of patients who present with metastatic foci to the thyroid tend to be older when compared to patients who have the usual metastatic foci, and they are often diagnosed with multiple malignancies.

Unlike colonic masses, the thyroid gland is easily accessible to assessment with routine physical exam and ultrasound. Fine needle aspiration (FNA) allows for pathologic diagnosis [12]. A large cytologic study consisting of 25,000 FNAs preformed on thyroid nodules identified an overall incidence of 0.1% to be metastatic neoplastic disease [13]. Preoperative distinction between a primary versus a secondary thyroid neoplasm can be difficult using cytology alone [14], and a thyroidectomy is frequently performed.

The most common initial metastatic location of colorectal cancer is the locoregional lymph nodes followed by the liver and the lungs. Seeding of the thyroid gland requires bypassing the portal circulation and entering into the systemic circulation. The exact mechanism of how metastases from colorectal cancer reach the thyroid gland is unclear. One may hypothesize that these cancers reach the thyroid because of their aggressive natures. Our patient did not seem to fit into the aggressive category compared to other patients with the same diagnosis. However, the thyroid with its rich blood flow of 560 ml/100g tissue/min [8] may be a logical location where these metastatic lesions could lodge. This high metabolic demand seems to be both protective and detrimental. It allows the gland to come in direct contact with blood borne neoplastic cells, leading to the potential for seeding. Alternatively, the high volume of blood flow through the gland potentially may lead to an immunoprotective environment in the thyroid, compared to the liver and lungs [15]. Yet, most of these metastases to the thyroid tend to remain subclinical or small in size compared to liver and lung metastases, which brings up the hypothesis that the thyroid environment is not as hospitable for the growth of metastatic neoplastic cells when compared to the liver and lungs. This may explain the disparity in low numbers of patients presented in the literature with a diagnosis of metastatic disease to the thyroid compared to autopsy results (1% - 3%) [6, 16]. Metastatic disease to the thyroid is believed to be a late event, almost always diagnosed years after the diagnosis of the initial primary disease [17]. Osin et al described a patient who had widely metastatic disease of the colon (stage IV) with large lesions within the hepatic parenchyma and only microscopic metastases to the thyroid gland [18]. Poon et al described a patient with primary colon carcinoma and metastases to the thyroid leading to locally compressive symptoms in the neck and ultimately leading to the overall demise of the patient [19]. Youn et al described a patient with colonic adenocarcinoma who had thyroid metastases diagnosed after developing clinical hypothyroidism [20].

Even though metastatic disease to the thyroid gland is a rare event, the concomitant high incidence of thyroid cancer makes these findings significant. The compilation of colon, renal and thyroid carcinomas gives rise to a rare and unique clinical situation. The available literature has not identified these three organs to be associated with a known neoplastic syndrome. Yet, despite the lack of reported data, one may infer that the thyroid gland is potentially a significant site for metastatic diseases.
